# Genomic Insights Into the *Treponema* Genus: Taxonomic Resolution of *Treponema vincentii* and Description of Two Novel Species, *Treponema plautii* sp. nov. and *Treponema sinense* sp. nov.

**DOI:** 10.1111/omi.70009

**Published:** 2025-09-12

**Authors:** Jordan Y. H. Fong, Man Lung Yeung, Tsz Tuen Li, Wing Ho Li, Yuanchao Ma, Yan Zhao, Wai Keung Leung, Jade L. L. Teng

**Affiliations:** ^1^ Faculty of Dentistry The University of Hong Kong Hong Kong Special Administrative Region China; ^2^ Department of Microbiology School of Clinical Medicine Li Ka Shing Faculty of Medicine The University of Hong Kong Hong Kong Special Administrative Region China; ^3^ State Key Laboratory of Emerging Infectious Diseases Li Ka Shing Faculty of Medicine The University of Hong Kong Hong Kong Special Administrative Region China; ^4^ Department of Clinical Microbiology and Infection Control The University of Hong Kong‐Shenzhen Hospital Shenzhen China; ^5^ Carol Yu Centre for Infection Li Ka Shing Faculty of Medicine The University of Hong Kong Hong Kong Special Administrative Region China

**Keywords:** 16S rDNA, genome sequencing, novel species, oral spirochetes, periodontal disease, *Treponema*

## Abstract

**Objectives:**

Periodontal disease, a global health concern, is strongly associated with oral treponemes. However, the taxonomy of some species remains unresolved, hindering our understanding of their roles in disease. This study aims to clarify the taxonomy of three strains isolated from patients with periodontal disease using phylogenomic and comparative genomic analyses.

**Materials and Methods:**

We performed genome sequencing for OMZ 800 and conducted phylogenomic and comparative genomic analyses of multiple strains to clarify their taxonomy.

**Results:**

Phylogenomic and in‐silico genome comparisons confirmed OMZ 800 as “*T. vincentii*” (Average Nucleotide Identity [ANI]>95%). We designated OMZ 800^T^ as the type strain for *T. vincentii* to establish its official standing in bacterial taxonomy. OMZ 806 (2.7 Mb, 44.9% GC) clustered with phylogroup IB strain (ANI>95% vs. OMZ 305), whereas OMZ 838 (2.7 Mb, 44.6% GC) clustered with phylogroup IA strains (ANI>95% vs. OMZ 855 and OMZ 857). Both OMZ 806 and OMZ 838 strains showed ANI<95% compared to *T. medium* ATCC700293^T^, supporting their classification as novel species. We propose *Treponema plautii* sp. nov. OMZ 806^T^ (with OMZ305 as additional strain) and *Treponema sinense* sp. nov. OMZ 838^T^ (with OMZ 855 and OMZ 857 as additional strains).

**Conclusion:**

This study clarifies *Treponema* taxonomy by designating OMZ 800^T^ as the type strain of *T. vincentii* and proposing two novel species, providing a refined taxonomical framework for this important genus.

## 1 Introduction

The human oral cavity is estimated to harbor over 500 bacterial species (W. E. Moore and Moore 1994). Although most of these bacteria are commensals, certain members can contribute to the development of periodontal diseases (How et al. 2016; Sela 2001). *Treponema*, commonly known as treponemes, are a prominent group of spirochete bacteria that inhabit the human oral cavity and are associated with periodontitis (Segata et al. 2012). Treponemes require a low‐oxygen environment, particularly within dental plaque biofilms in the gingival sulcus, a shallow crevice of gum tissue surrounding the base of the tooth (Dashper et al. 2011; Ruby et al. 2018). In individuals with periodontal diseases, such as gingivitis and periodontitis, treponeme numbers are often significantly higher in subgingival sites (Armitage et al. 1982; Ellen and Galimanas 2005; Listgarten and Hellden 1978; Loesche et al. 1985; L. V. Moore et al. 1987; Paster et al. 2001). Despite comprising less than 2% of the healthy human oral microbiome, the diversity of treponemes plays a significant role in the development and progression of periodontitis (Segata et al. 2012; Zeng et al. 2021).

Currently, the genus *Treponema* comprises 28 validated species names included in the List of Prokaryotic Names with Standing in Nomenclature (Parte et al. 2020). Of these, nine *Treponema* species are of oral origins and associated with periodontal diseases: *T. amylovorum*, *T. denticola*, *T. lecithinolyticum*, *T. maltophilum*, *T. medium*, *T. parvum*, *T. pectinovorum*, *T. putidum*, and *T. socranskii* (E. C. Chan et al. 1993; Smibert and Burmeister 1983; Smibert et al. 1984; Wyss et al. 1996, 1997, 1999). Based on 16S rDNA sequencing, a previous study reported over 75 phylotypes in the human oral cavity, suggesting the presence of a large uncultivated population of oral treponemes that warrants further investigation (Huo et al. 2017). This further classifies human oral treponemes into 10 primary phylogroups designated as 1 to 10 (Dewhirst et al. 2000; Huo et al. 2017; Zeng et al. 2021). Subsequent research has associated nine *Treponema* species with distinct phylogroups: *T. medium* (phylogroup 1), *T. denticola* and *T. putidum* (phylogroup 2), *T. lecithinolyticum* and *T. maltophilum* (phylogroup 4), *T. amylovorum* (phylogroup 5), *T. socranskii* (phylogroup 6), *T. parvum* (phylogroup 7), and *T. pectinovorum* (phylogroup 8) (E. C. Chan et al. 1993; Smibert and Burmeister 1983; Smibert et al. 1984; Wyss et al. 1996, 1997, 1999). Notably, no species has yet been assigned to phylogroups 3, 9, or 10. Within phylogroup 1, the species “*T. vincentii*” has been extensively studied for over 60 years (Huo et al. 2017; Klein 1946), yet its type strain remains undefined and lacks official taxonomic status (E. C. Chan and McLaughlin 2000; Huo et al. 2017). Beyond 16S rDNA sequencing, multi‐locus sequence analysis (MLSA) has been used to classify *Treponema* species with increased resolution (Huo et al. 2017). For instance, the analysis of four highly conserved genes (16S rDNA, *recA*, *pyrH*, and *flaA*) successfully classified two treponemal isolates from phylogroup 1 into phylotypes IA and IB, potentially representing novel species (Y. Chan et al. 2014; Huo et al. 2017; Wyss 1998; You et al. 2013; Zeng et al. 2021). These two treponemal isolates, OMZ 806 and OMZ 838, have been referred to as *T. medium*‐like and “*T. vincentii*”‐like, respectively, with limited information available in the literature (Y. Chan et al. 2014; Huo et al. 2017; Zeng et al. 2021).

Accurate taxonomic descriptions of *Treponema* species are essential for understanding their roles in periodontal diseases and the oral microbiome (You et al. 2013; Zeng et al. 2021). Despite significant research updates, some oral treponeme species still lack well‐defined taxonomic classifications, hindering our understanding of their importance in dentistry (Y. Chan et al. 2014; Zeng et al. 2021). Therefore, this study aims to clarify the taxonomic status of three *Treponema* species with ambiguous taxonomic status using a phylogenomic approach. These species include *Treponema* sp. OMZ 838 and *Treponema* sp. OMZ 806, isolated from patients with periodontitis and acute necrotizing ulcerative gingivitis (NUG) in China and Europe, respectively (Y. Chan et al. 2014; Zeng et al. 2021), as well as “*T. vincentii*”, which has been extensively studied due to its important role in oral diseases (Brumpt 1922; Edwards et al. 2003; Paster 2010; Zeng et al. 2021). The “*T. vincentii*” OMZ 800 genome was sequenced, and comparative genomic analyses were conducted with *Treponema* sp. OMZ 806, *Treponema* sp. OMZ 838, and additional representative *Treponema* species. Based on the findings, we propose a new taxonomic status for “*T. vincentii*” with the designation of OMZ 800^T^ as its type strain, and the naming of *T. plautii* OMZ 806^T^ sp. nov. and *T. sinense* OMZ 838^T^ sp. nov. as two novel species within the *Treponema* genus. This taxonomic clarification will enhance our understanding of these *Treponema* species and their implications in oral health and disease.

## 2 Materials and Methods

### 2.1 Bacterial Strains

“*T. vincentii*” OMZ 800, *Treponema* sp. OMZ 806, and *Treponema* sp. OMZ 838 were originally isolated in 1998 by C. Wyss (University of Zurich). “*T. vincentii*” OMZ 800 and OMZ 806 were isolated from patients with periodontitis (Wyss 1998), whereas OMZ 838 was isolated from a microbial biofilm originally sampled from a NUG lesion in the oral cavity of a Chinese male from Northeast China (Y. Chan et al. 2014).

### 2.2 Bacterial Culture

Details of the oral treponeme strains used in this study are summarized in Table S1. Strains of “*T. vincentii*” OMZ 800 (ATCC 700765, DSMZ 16788), *Treponema* sp. OMZ 806 (ATCC 700767, DSMZ 16787), and *Treponema* sp. OMZ 838 (ATCC 700772, DSMZ 16789) was purchased from the Leibniz Institute DSMZ. The strains were stored at −80°C and cultivated anaerobically in TYGVS medium supplemented with thiamine pyrophosphate (Sigma‐Aldrich, USA), volatile fatty acids (Sigma‐Aldrich), and 10% fetal bovine serum (Gibco, USA) as described previously (Huo et al. 2017).

### 2.3 Genome Sequencing and Assembly

Since no genome sequence was available for the strain “*T. vincentii*” OMZ 800, therefore, its genome was sequenced using Illumina technology. The strain “*T. vincentii*” OMZ 800 was incubated anaerobically at 37°C for 7 days until active growth became apparent. The cells were then harvested by centrifugation (13,000 rpm; 5 min) and washed with 5 mL of phosphate‐buffered saline for DNA extraction using a genomic DNA purification kit (QIAgen, Germany). The Illumina DNA library was prepared using a Nextera XT DNA Sample Prep Kit (Illumina, USA) and sequenced on a NovaSeq 6000 instrument (run type: PE151 bp). The resulting FASTQ reads were processed using fastp to filter low‐quality reads and trim adapter sequences (Chen 2023). The filtered reads were then assembled using SPAdes to generate a draft genome of “*T. vincentii*” OMZ 800 (Bankevich et al. 2012; Teng et al. 2021).

### 2.4 Genome Sequence Analyses

A total of 32 genomes were analyzed in this study, representing 22 known *Treponema* species and two novel species. Except for the OMZ 800^T^, which was sequenced as part of this study, the remaining 31 genome sequences from 22 *Treponema* species were retrieved from the GenBank database (Table S1). The analyzed genomes included “*T. vincentii*” OMZ 800, *Treponema* sp. OMZ 806, and *Treponema* sp. OMZ 838 (*n* = 3), additional strains of the studied species (*n* = 5), and type strains of other representative *Treponema* species (*n* = 22) (Table S1). For genome characterization and annotation, the DDBJ Fast Annotation and Submission Tool (DFAST) and Rapid Annotations using Subsystems Technology (RAST) were employed to conduct genomic characterizations and functional annotations to identify genome features, protein‐coding genes, as well as potential antibiotic resistance genes and virulence genes (Aziz et al. 2008; Tanizawa et al. 2018). The Kruskal–Wallis test was used to assess statistical differences in functional annotations across the study isolates. The Proksee server was used to generate circular genomes of three *Treponema* isolates (Grant et al. 2023). For circular genome annotation, the Prokka plugin was used with default parameters to determine the coding sequences (CDS), tRNAs, repeat regions, and rDNAs (Grant et al. 2023; Seemann 2014). Sequence composition functions, including GC content and skew, were calculated with a window size of 10,000 bp and a step size of 100 bp, respectively.

Digital DNA–DNA Hybridization (dDDH) and Average Nucleotide Identity (ANI) were calculated to determine the intergenomic distance between OMZ 800, OMZ 806, and OMZ 838, as well as the existing 24 type strain genomes from 22 *Treponema* species, and five additional strains of the studied species. These analyses were performed using the OrthoANI via the Type (Strain) Genome Server (TYGS) pipeline (https://tygs.dsmz.de/) (Lee et al. 2016; Meier‐Kolthoff et al. 2022). The intergenomic distance threshold values of 70% (dDDH) and 95% (ANI) were used to differentiate between *Treponema* species and delineate boundaries for same‐species classification.

### 2.5 Phylogenetic Characterization

Comparative sequence analyses based on the 16S rDNA sequence and genome were conducted to determine the phylogenetic position of “*T. vincentii*” OMZ 800, *Treponema* sp. OMZ 806, and *Treponema* sp. OMZ 838 among the current 22 species within the genus *Treponema* (Pan et al. 2018).

The 16S rDNA sequences were extracted using BAsic Rapid Ribosomal RNA Predictor (barrnap) after genome assembly (Seemann 2013). The DNA alignment was performed using the MUSCLE alignment tool with the default parameters (Edgar 2004). The aligned DNA was then imported into MEGA 11 (version 11.0.11) to create a phylogenetic tree using the Maximum‐Likelihood (ML) method (Tamura et al. 2021). The best substitution model, Tamura‐Nei with gamma distribution and an evolutionarily invariable (TN93 + G + I), was selected for creating the ML tree based on the lowest Bayesian Information Criterion (BIC) scores. The neighbor‐joining tree was also constructed using MEGA 11 (version 11.0.11) (Tamura et al. 2021).

A phylogenomic tree was constructed using the TYGS pipeline with 32 genome sequences from 22 *Treponema* species to determine the taxonomic positions of “*T. vincentii*” OMZ 800, *Treponema* sp. OMZ 806, and *Treponema* sp. OMZ 838 (Meier‐Kolthoff et al. 2022). The whole‐genome sequence‐based (GBDP) tree was then exported for manual annotation.

## 3 Results

### 3.1 Genome Characteristics of “*T. vincentii*” OMZ 800, *Treponema* sp. OMZ 806, and *Treponema* sp. OMZ 838

**FIGURE 1:**
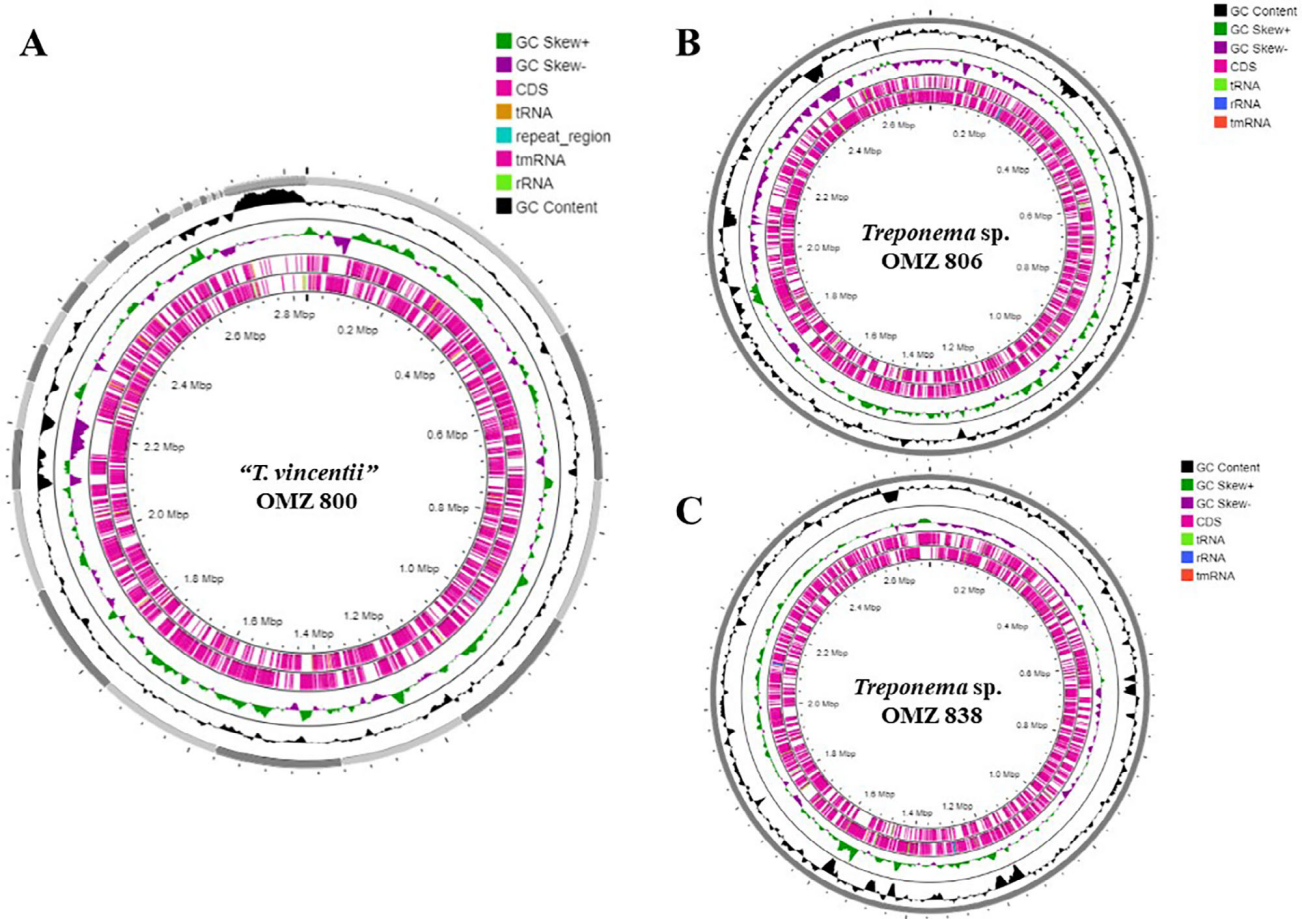
Circular genome representations of three proposed species: A. “*T. vincentii*” OMZ 800, B. *Treponema* sp. OMZ 806, and C. *Treponema* sp. OMZ 838. The plots, generated using Proskee, show the characteristic features of each genome from the outside to the inner of the circle. The rings represent GC skew, CDSs, tRNAs, repeat regions, and GC content, are indicated by color coding.

Genomic characterizations and comparisons between “*T. vincentii*” OMZ 800, *Treponema* sp. OMZ 806, and *Treponema* sp. OMZ 838 and other *Treponema* species were studied to obtain a deeper understanding of the genome characteristics and taxonomic relationships of these three isolates (Tables 1 and 2; Figure 1). Apart from the genome of “*T. vincentii*” OMZ 800, which was sequenced in the current study, the remaining genomes were retrieved from GenBank (Table S1). We also included five additional strains from *Treponema* phylogroup 1 (*n* = 5), which were previously reported as distinct phylotypes based on MLSA: “*T. vincentii*” ATCC35580 and “*T. vincentii*” F0403 (phylogroup 1), *Treponema* sp. OMZ 855 and *Treponema* sp. OMZ 857 (phylotype IA), and *Treponema* sp. OMZ 305 (phylotype IB) (Huo et al. 2017).

**TABLE 1:** Genome characteristics between *Treponema* species included in the analysis of this study.

*Treponema* species	Number of contigs	N50	Total sequence length (bp)	GC content (%)	Number of CDSs	Average protein length	Coding ratio (%)	Number of rDNA	Number of tRNAs	Number of CRISPRs
*Treponema* species of human origin										
Phylogroup 1										
“*T. vincentii*” OMZ 800	35	199,968	2,829,797	46.5	2352	340	85	3	50	3
*Treponema* sp. OMZ 806	1	2,711,688	2,711,688	44.9	2539	311	87	6	50	0
*Treponema* sp. OMZ 838	1	2,708,067	2,708,067	44.6	2437	330	89	6	49	0
*T. medium* ATCC 700293^T^	1	2,727,508	2,727,508	44.3	2313	339	86	5	49	1
*Treponema* sp. IA OMZ 855	1	2,629,551	2,629,551	44.5	2271	340	88	6	49	0
*Treponema* sp. IA OMZ 857	1	2,689,385	2,689,385	44.3	2393	335	90	6	50	0
*T Treponema* sp. OMZ 305	1	2,842,704	2,842,704	45.1	2547	331	89	6	53	1
*T. vincentii* ATCC 35580	79	114,980	2,514,590	45.7	2274	320	87	3	49	2
*T. vincentii* F0403	3	2,347,197	2,693,493	45.5	2415	330	89	5	48	0
Phylogroup 2^a^	1.5 ± 0.7	2,820,057 ± 32,731	2,821,881.5 ± 30,150.3	37.6 ± 0.4	2603.5 ± 30.4	329 ± 9.7	91 ± 0.6	6 ± 0	44.5 ± 0.7	1 ± 0
Phylogroup 4^b^	11.0 ± 14.1	1,471,161 ± 1,497,973.189	2,435,636.5 ± 133,998.9	45.8 ± 2.9	2156.5 ± 167.6	347.7 ± 11.3	92.2 ± 0.9	4.5 ± 2.1	45 ± 1.4	3 ± 1.4
Phylogroup 6^c^	25.7 ± 41.9	1,637,603 ± 1,395,203	2,784,172.7 ± 36,397.8	48.9 ± 0.6	2441.7 ± 21.5	336.6 ± 2.7	88.6 ± 0.3	7 ± 3.5	47.3 ± 1.2	2.3 ± 0.6
Phylogroup 7										
*T. parvum* OMZ 833^T^	1	2,609,480	2,609,480	44.4	2309	331.3	88	6	47	1
Phylogroup 8										
*T. pectinovorum* ATCC 700769	6	1,756,681	2,319,274	36.9	2021	345.1	90.2	3	42	2
Unassigned										
*T. peruense* RCC2812^T^	1	2,738,066	2,738,066	41.2	2434	341.2	91	14	52	1
*Treponema* species of animal origin^d^	14.8 ± 18.3	1,838,707 ± 1,613,868	3,176,266 ± 480,758.6	43.9 ± 5.8	2759.2 ± 424.5	344.9 ± 16.3	89.8 ± 2.8	5.5 ± 3.6	48.1 ± 6	1.4 ± 2

*Note*: Numbers represent average and standard deviation of annotated genome features determined by dFAST.

^a^ Phylogroup 2 includes *T. denticola* ATCC 35405^T^ and *T. putidum* OMZ 758^T^.

^b^ Phylogroup 4 includes *T. lecithinolyticum* ATCC 700332^T^ and *T. maltophilum* ATCC 51939^T^.

^c^ Phylogroup 6 includes *T. socranskii* subsp. *buccale* ATCC 35534^T^, *T. socranskii* subsp. *paredis* ATCC 35535^T^, and *T. socranskii* subsp. *socranskii* ATCC 35536^T^.

^d^ Animal origin *Treponema* includes *T. azotonutricium* ZAS‐9^T^, *T. berlinense* ATCC BAA‐909^T^, *T. brennaborense* DSM 12168^T^, *T. bryantii* NK4A124^T^, *T. pedis* T3552B^T^, *T. phagedenis* B43.1^T^, *T. porcinum* ATCC BAA‐908^T^, *T. primitia* ZAS‐2^T^, *T. rectale* DSM 103679^T^, *T. ruminis* DSM 103462^T^, *T. saccharophilum* DSM 2985^T^, *T. succinifaciens* DSM 2489^T^.

**TABLE 2:** ANI and dDDH values as calculated by OrthoANI and TYGS with “*T. vincentii”* OMZ 800^T^, *Treponema* sp. OMZ 806, *Treponema* sp. OMZ 838 as query against other *Treponema* strains as reference. The values are sorted in descending order based on ANI, and the top 10 closest strains are shown.

Query strain	Subject strain	ANI %	dDDH (d4, in %)^a^
“*T. vincentii*” OMZ 800	“*T. vincentii*” ATCC 35580	97.9	80
	“*T. vincentii*” F0403	97.8	80.2
	*Treponema* sp. OMZ 838	85.9	30.7
	*T. medium* ATCC 700293^T^	85.7	30.8
	*Treponema* sp. OMZ 806	85.5	30.3
	*Treponema* sp. IA OMZ 855	85.5	30.7
	*Treponema* sp. OMZ 305	85.5	30.3
	*T. maltophilum* ATCC 51939^T^	68.6	31.3
	*T. putidum* OMZ 758^T^	68.2	26.5
	*T. denticola* ATCC 35405^T^	68.1	24.2
*Treponema* sp. OMZ 806	*Treponema* sp. OMZ 305	96.4	70.2
	*T. medium* ATCC 700293^T^	91.7	44.1
	*Treponema* sp. OMZ 855	89.9	38.5
	*Treponema* sp. OMZ 838	89.8	38.8
	*Treponema* sp. OMZ 857	89.7	38.4
	“*T. vincentii*” OMZ 800	85.5	30.3
	“*T. vincentii*” ATCC 35580	85.4	30
	“*T. vincentii*” F0403	85.3	30.1
	*T. denticola* ATCC 35405^T^	69.5	31.1
	*T. maltophilum* ATCC 51939^T^	69.1	34.9
*Treponema* sp. OMZ 838	*Treponema* sp. OMZ855	98.1	81.6
	*Treponema* sp. OMZ857	97	72.2
	*T. medium* ATCC 700293^T^	90.9	42.1
	*Treponema* sp. OMZ 806	89.8	38.8
	*Treponema* sp. OMZ305	89.8	38.7
	“*T. vincentii*” F0403	85.8	31.3
	“*T. vincentii*” ATCC 35580	85.8	31
	“*T. vincentii*” OMZ 800	85.7	30.7
	T. *maltophilum* ATCC 51939^T^	69.7	39.1
	*T. denticola* ATCC 35405^T^	69.7	31

^a^ The computations are based on dDDH formula d4 which is equivalent to GGDC formula 2.

The genome of “*T. vincentii*” OMZ 800 was sequenced, generating 9,266,734 raw reads, of which 9,217,259 (99.4%) remained after quality filtering. This dataset provided coverage exceeding 400×, enabling the assembly of 35 contigs with an N50 value of 199,968 bp. The assembled genome has a predicted size of 2,829,797 bp and a GC content of 46.5%. Comparative genomic analysis revealed conserved features across “*T. vincentii*” isolates, including OMZ 800, ATCC 35580, and F0403 (Table 1). All three strains demonstrated near‐identical coding potential, with closely matched CDS counts (2,274–2,415), average protein lengths (320–340 residues), and coding ratios (85%–89%).  “*T. vincentii*” OMZ 800 and ATCC 35580 shared three copies of rDNA, whereas F0403 harbored five rDNA copies. Conversely, tRNA copy numbers were highly conserved, ranging from 48 to 49 across all isolates. No CRISPR arrays were detected in F0403, whereas ATCC 35580 and OMZ 800 harbored two and three CRISPR loci, respectively.

The genomic architecture of *Treponema* sp. OMZ 806 and OMZ 305 exhibited strong conservation in characteristics. Genome sizes were comparable at 2,711,688 bp (OMZ 806) and 2,842,704 bp (OMZ 305), with similar GC contents of 44.9% and 44.3%, respectively (Table 1). Both strains shared similar coding capacities, with 2539 and 2547 CDS in OMZ 806 and OMZ 305, respectively, exhibiting average protein lengths (311.3 in OMZ 806 vs. 331.2 amino acids in OMZ 305) and coding ratios (87.4% in OMZ 806 vs. 89% in OMZ 305). Both strains harbored six rDNA copies and similar tRNA copy numbers (50 in OMZ 806 vs. 53 in OMZ 305). However, differences were observed in CRISPR elements between the two strains, with OMZ 806 lacking CRISPR sequences, whereas OMZ 305 retained one CRISPR array, implying differential evolutionary exposure to mobile genetic elements.

The genomes of *Treponema* sp. OMZ 838, OMZ 855, and OMZ 857 are also highly similar. OMZ 838 contained a 2,708,067 bp genome with a 44.6% GC content, whereas OMZ 855 and OMZ 857 had 2,629,551 bp (44.5% GC) and 2,689,385 bp (44.3% GC), respectively, reflecting limited intra‐phylotype variation (Table 1). All three strains demonstrated similar coding potential, with similar CDS counts (2271–2437), average protein lengths (330–340 residues), and coding ratios (88%–90%). Each isolate harbored six rDNA sequences and similar tRNA copy numbers (49–50), whereas CRISPR arrays were absent in all strains, highlighting evolutionary conservation within this phylotype.

### 3.2 Sequence and Phylogenetic Analysis Based on 16S rDNA

**FIGURE 2:**
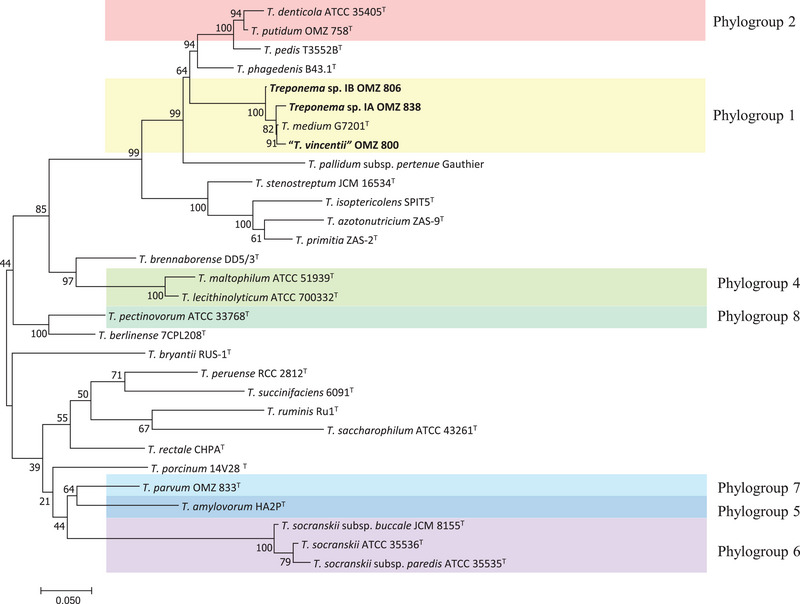
Phylogenetic tree based on 16S rDNA sequence of “*T. vincentii*” OMZ 800, *Treponema* sp. OMZ 806, and *Treponema* sp. OMZ 838, along with all currently recognized species of the genus *Treponema*. The evolutionary history was inferred using the Maximum Likelihood method and the Tamura‐Nei model. The percentage of trees in which the associated taxa clustered together is shown next to the branches (bootstrap support). The tree is drawn to scale, with branch lengths measured in the number of substitutions per site. This analysis involved 30 nucleotide sequences. The strains studied in this work are highlighted in bold. Evolutionary analyses were conducted in MEGA11. The 16S rDNA sequences of “*T. vincentii*” OMZ 800, *Treponema* sp. OMZ 806, and *Treponema* sp. OMZ 838 were extracted from their respective genomes. Details of the studied strains and their accession numbers are provided in Table S1.

Phylogenetic analysis of 16S rDNA sequences from “*T. vincentii*” OMZ 800, *Treponema* sp. OMZ 806, and *Treponema* sp. OMZ 838, along with genomes of validated *Treponema* species, was used to establish the evolutionary relationships. The analysis revealed that all three isolates clustered together with 100% bootstrap support (Figure 2). Furthermore, the three isolates were grouped with *T. medium* (phylogroup 1), a validated species closely associated with “*T. vincentii*”.

The 16S rDNA gene sequence identities for “*T. vincentii*” OMZ 800, *Treponema* sp. OMZ 806, and *Treponema* sp. OMZ 838 with their closest relative, *T. medium* GT201^T^, were 98.7%, 98.1%, and 98.5%, respectively. Conversely, when compared to *T. denticola* and *T. putidum* (Phylogroup 2), the sequence identities were less than 90.8% and 91.4%, respectively. Further comparison of the 16S rDNA sequences among “*T. vincentii*” OMZ 800, *Treponema* sp. OMZ 806, and *Treponema* sp. OMZ 838 showed interspecies identities ranging from 98.3% to 98.7%, suggesting that the 16S rDNA sequence alone may be insufficient to differentiate between these *Treponema* species.

### 3.3 Phylogenomic Analysis

**FIGURE 3:**
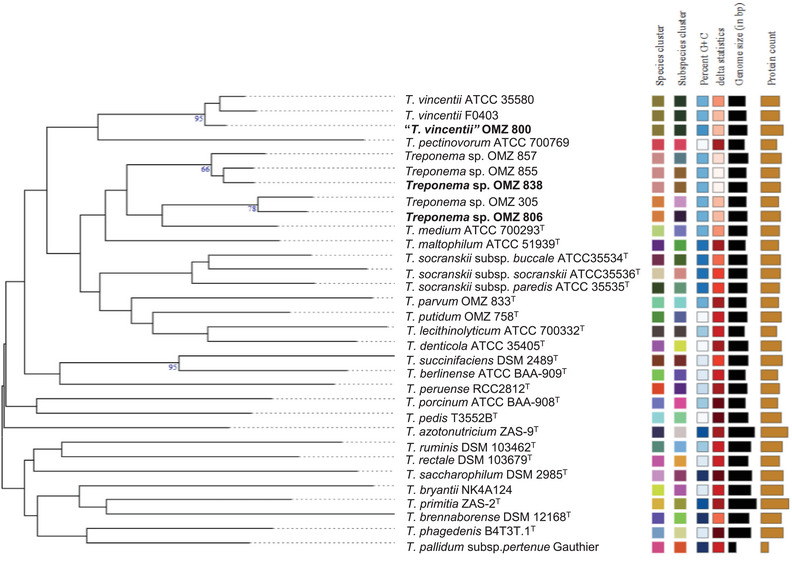
TYGS‐generated phylogenetic tree illustrating the relationships between strains “*T. vincentii*” OMZ 800, *Treponema* sp. OMZ 806, and *Treponema* sp. OMZ 838, along with related type strains and non‐validated *Treponema* isolates. The numerical values above the branches represent GBDP pseudo‐bootstrap support values exceeding 60% from 100 replications. Leaf labels are annotated with species and subspecies clusters, genomic GC content, *δ* values, overall genome size, and number of proteins.

The phylogenomic tree, constructed using TYGS GBDP distance calculations, revealed that “*T. vincentii*” OMZ 800 clustered closely with “*T. vincentii*” F0403 and “*T. vincentii*” ATCC35580, supported by a bootstrap value of 95% (Figure 3). Furthermore, *Treponema* sp. OMZ 806 was found to cluster with *Treponema* sp OMZ 305, a member of phylogroup IB (Figure 3). *Treponema* sp. OMZ 838 grouped with two phylogroup IA isolates (*Treponema* sp. OMZ 855 and *Treponema* sp. OMZ 857), forming a distinct species clade (Figure 3). These findings highlight the distinct clustering patterns among the studied isolates, suggesting potential taxonomic differentiation within the *Treponema* genus.

### 3.4 Intergenomic Analysis

In silico genome‐to‐genome comparisons were conducted with OrthoANI and the TYGS server to calculate the ANI and dDDH values, respectively. The OrthoANI analysis showed that “*T. vincentii*” OMZ 800 exhibited the highest similarity to “*T. vincentii*” ATCC35580 [ANI of 97.9%], followed by “*T. vincentii*” F0403 [ANI of 97.8%]. These ANI values exceeded the established 95% ANI threshold for species delineation, confirming the classification of OMZ 800 within the “*T. vincentii*” species (Table 2). Therefore, we propose OMZ 800^T^ as the type strain for *T. vincentii*.

For *Treponema* sp. OMZ 806, the closest relative was *Treponema* sp. OMZ 305 [ANI of 96.4%], followed by *T. medium* ATCC 700293^T^ [ANI of 91.7%]. These results suggest that OMZ 806 and OMZ 305 belong to the same species and should be classified as a novel *Treponema* species. We tentatively named this novel species *T. plautii* sp. nov., with OMZ 806^T^ proposed as the type strain and OMZ 305 recognized as an additional strain.

For *Treponema* sp. OMZ 838, the closest relative was *Treponema* sp. OMZ 855 [ANI of 98.1%], followed by *Treponema* sp. OMZ 857 [ANI of 97%], and *T. medium* ATCC 700293^T^ [ANI of 90.9%]. These results suggest that OMZ 838, OMZ 855, and OMZ 857 belong to the same species, and they should be classified as a novel *Treponema* species. We tentatively named this novel species *T. sinense* sp. nov., with OMZ 838^T^ proposed as the type strain and OMZ855 and OMZ857 recognized as additional strains.

An independent genome‐to‐genome comparison using dDDH via the TYGS platform was conducted. The results were consistent with the ANI data (Table 2), confirming that OMZ 800^T^ corresponds to the *T. vincentii* species, whereas *T. plautii* OMZ 806^T^ and *T. sinense* OMZ 838^T^ represent novel *Treponema* species that have not been previously described.

### 3.5 Functional Annotation of Protein‐Coding Genes

**FIGURE 4:**
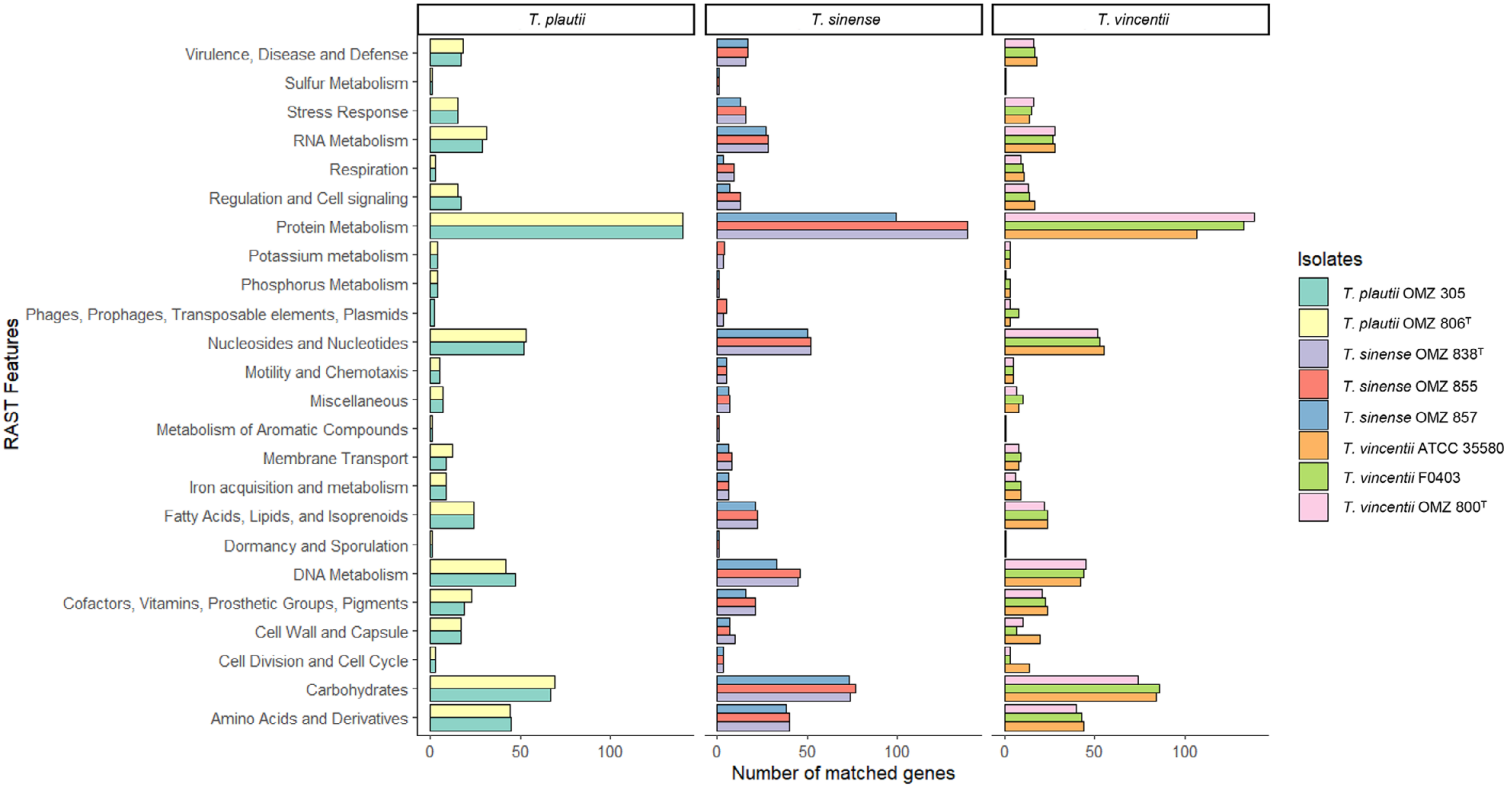
Functional annotation summary of *T. vincentii* (OMZ 800^T^; F0403; ATCC 35580), *T. plautii* (OMZ 806^T^; OMZ 305), and *T. sinense* (OMZ 838^T^; OMZ 855; OMZ 857). After gene calling, the RAST server assigned protein‐coding genes to 24 metabolic pathways via the subsystem technology.

Functional annotation of protein‐coding genes in *T. vincentii* OMZ 800^T^, *T. plautii* OMZ 806^T^, and *T. sinense* OMZ 838^T^, as well as some additional strains, revealed the genomic diversity and functional potential of *Treponema* isolates. Comparative analysis across isolates showed diverse gene counts in different functional categories (Figure 4). However, no statistically significant differences (*p* > 0.05) were observed between the species based on the Kruskal–Wallis test, indicating a degree of functional similarity among the *Treponema* isolates.

The RAST analysis identified conserved categories, such as “Dormancy and Sporulation” and “Sulfur Metabolism”, which were each represented by a single gene across all isolates. “Motility and Chemotaxis” exhibited consistent annotation with five genes in all isolates, suggesting the preservation of essential biological functions. Conversely, gene counts were variable in categories, including “Amino Acids and Derivatives” (38–45 genes), “Carbohydrates” (67–86 genes), “Membrane Transport” (6–12 genes), “Protein Metabolism” (99–140 genes), and “Phages, Prophages, Transposable elements”, and “Plasmids” (0–8 genes), suggesting functional diversity within these pathways. The broad variability in gene counts, particularly in “Protein Metabolism”, underscores the complexity of metabolic processes in *Treponema* (Figure 4).

## 4 Discussion

The presence of abundant oral spirochete bacteria within periodontal pockets is consistently associated with the occurrence and severity of periodontal disease (Loesche 1988; Simonson et al. 1988; You et al. 2013). Molecular approaches, such as 16S rDNA gene sequencing, have demonstrated that the resident oral spirochete community predominantly belongs to the genus *Treponema* (Dewhirst et al. 2000). However, a significant proportion of *Treponema* phylotypes remain unclassified at the species level due to cultivation limitations, resulting in a limited understanding of their taxonomic diversity and potential roles in periodontal pathogenesis (Dewhirst et al. 2000; Siqueira and Rocas 2013).

**FIGURE 5:**
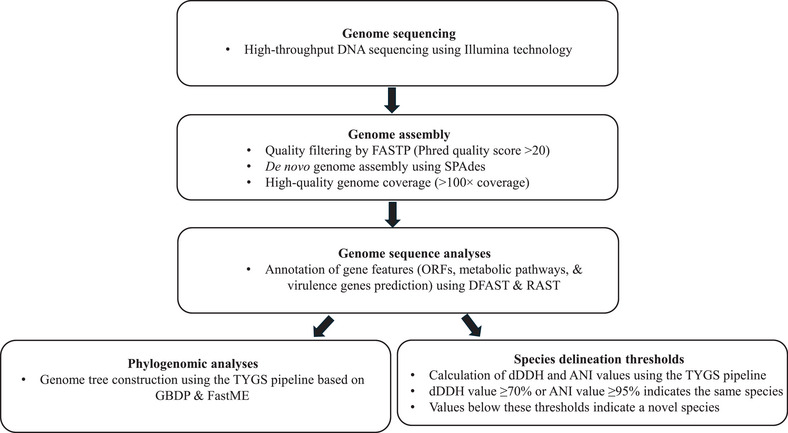
Flowchart summarizing the phylogenomic methodology used in this study to elucidate taxonomic relationships among *Treponema* species.

In this study, we employed comprehensive genomic characterization to resolve longstanding ambiguities regarding the definition of the type strain OMZ 800^T^ for *T. vincentii* and facilitated the formal recognition of two novel species, *T. plautii* sp. nov. and *T. sinense* sp. nov. (Figure 5). Comparative genomic analyses revealed that these three isolates belong to *Treponema* phylogroup 1, which is among the most prevalent in dental plaque microbiomes and has been linked to periodontitis, particularly refractory cases, and acute NUG (Dewhirst et al. 2000; You et al. 2013). Despite their close genomic relatedness to *T. medium*, each isolate represents a distinct species, underscoring the rich diversity within this phylogroup (Figures 2 and 3; Table 2).

The refinement of *Treponema* taxonomy through genome‐based approaches marks a significant advancement in our understanding of their diversity in the oral microbiome, encompassing both health‐ and disease‐associated states. This higher resolution enables more precise differentiation of strains, facilitating investigations into their specific contributions to oral homeostasis and disease processes. Moreover, insights into their ecological niches, virulence factors, and antimicrobial resistance profiles are crucial for elucidating the mechanisms that underpin microbial balance and dysbiosis within the oral cavity. For instance, identifying species with heightened virulence or resistance traits can inform targeted antimicrobial therapies and microbiome modulation strategies.

Overall, the integration of genomic data into *Treponema* taxonomy broadens our understanding of the microbial landscape associated with oral health and disease and provides a foundation for developing improved diagnostic tools and personalized therapeutic interventions. As genomic technologies continue to advance, their application in oral microbiome research holds tremendous potential for early detection, targeted treatment, and effective management of periodontal diseases, contributing to more personalized and effective oral healthcare.

## 5 Conclusion

This study resolves taxonomic ambiguities within oral *Treponema* by designating *T. vincentii* OMZ 800^T^ as the definitive type strain and proposing two novel *Treponema* species: *Treponema plautii* sp. nov. (with OMZ 806^T^ as the type strain and OMZ 305 as an additional strain) and *Treponema sinense* sp. nov. (with OMZ 838^T^ designated as the type strain, as well as OMZ855 and OMZ857 as additional strains). These findings, based on genome‐based criteria (ANI/dDDH), advance the taxonomic framework for enhancing our understanding of spirochete diversity and evolutionary relationships.

## 6 Taxonomy

### 6.1 Proposal of OMZ 800^T^ as the type strain for *T. vincentii*

*T. vincentii* (vin. cen’. ti. i. L. gen. masc. n. *vincentii*, of Vincent, referring to H. Vincent who studied the organism originally isolated from Vincent's angina and NUG) (Dzink et al. 1990; Yue et al. 2023).

Cells are gram‐negative, motile, obligatory anaerobic oral spirochetes. Under fluorescent microscopy, cells are motile spirochetes with a helical coil and exhibit jerky flexing. Cells are approximately 5–10 µm long and 0.15–0.25 µm wide, with two to five irregular spirals and four periplasmic flagella attached at each end in a 4:8:4 arrangement. Cells require a 48–72 h anaerobic incubation at 35°C in TYGVS medium supplemented with thiamine pyrophosphate, volatile fatty acids, and 10% rabbit serum. β‐hemolysis was not observed. API ZYM analysis identified positive reactions for C4 esterase, C8 esterase, lipase, and leucine arylamidase. Negative reactions were observed for α‐galactosidase, C14 lipase, valine arylamidase, cystine arylamidase, β‐glucuronidase, α‐glucosidase, β‐glucosidase, α‐mannosidase, and α‐fucosidase. The designation *Treponema vincentii* has no official standing in bacterial taxonomy despite its frequent use in literature. Historically, this name has been applied to a variety of treponemal isolates, leading to significant confusion regarding its identity and classification. In view of this ambiguous history, the strain OMZ 800^T^ was proposed as the type strain of *T. vincentii* to formally establish the taxonomic status of this extensively studied *Treponema* species. This assignment aims to clarify the taxonomy of this species, serving as a definitive reference point for future studies.

The type strain, OMZ 800^T^ (= DSM 16788^T^ = ATCC 700765^T^), was isolated from a patient with periodontitis. The GC content of the type strain OMZ 800^T^ DNA was 46.5%. The GenBank accession number of the strain OMZ 800^T^ genome is GCA_042465945.1.

### 6.2 Description of *T. plautii* sp. nov

*T. plautii* (plau'ti.i. N.L. gen. masc. n. *plautii* of Plaut, named in honor of the German physician H. C. Plaut, for his studies on trench mouth).

Cells are gram‐negative, motile, obligatory anaerobic oral spirochetes. Under fluorescent microscopy, spirochetes appear motile with a helical coil and irregular movements. Cells are approximately 6–12 µm long and 0.16–0.3 µm wide, with two to five irregular spirals and two periplasmic flagella attached at each end in a 2:4:2 arrangement. Cells require a 48–72 h anaerobic incubation at 35°C in TYGVS medium supplemented with thiamine pyrophosphate, volatile fatty acids, and 10% rabbit serum. β‐hemolysis was not observed. API ZYM analysis identified positive reactions for C4 esterase, C8 esterase, lipase, and leucine arylamidase. Negative reactions were observed for α‐galactosidase, C14 lipase, valine arylamidase, cystine arylamidase, β‐glucuronidase, α‐glucosidase, β‐glucosidase, α‐mannosidase, and α‐fucosidase.

The type strain, OMZ 806^T^ (= DSM 16787^T^ = ATCC 700767^T^), was isolated from a patient with periodontitis. The GC content of the type strain OMZ 806^T^ DNA was 44.9%.

### 6.3 Description of *T. sinense* sp. nov

*T. sinense* (si.nen′se. N.L. neut. adj. *sinense*, pertaining to China).

Cells are gram‐negative, motile, obligatory anaerobic spirochetes, indigenous to NUG lesions in humans. Under fluorescent microscopy, spirochete cells appear motile with a helical coil and irregular movements. Cells are approximately 5–11 µm long and 0.15–0.25 µm wide, with two to five irregular spirals and two periplasmic flagella attached at each end in a 2:4:2 arrangement. Cells require a 48–72 h anaerobic incubation at 35°C in TYGVS medium supplemented with thiamine pyrophosphate, volatile fatty acids, and 10% rabbit serum. β‐hemolysis was not observed. API ZYM analysis identified positive reactions for C4 esterase, C8 esterase, lipase, and leucine arylamidase. Negative reactions were observed for α‐galactosidase, C14 lipase, valine arylamidase, cystine arylamidase, β‐glucuronidase, α‐glucosidase, β‐glucosidase, α‐mannosidase, and α‐fucosidase.

The type strain, OMZ 838^T^ (= DSM 16789^T^ = ATCC 700772^T^), was isolated from a patient with an NUG lesion. The GC content of the type strain OMZ 838^T^ DNA was 44.6%.

## Author Contributions

Conceptualization, J. L. L. T. and W. K. L.; methodology, J. Y. H. F., T. T. L., W. H. L., Y. M and Y. Z.; investigation, J. L. L. T. and J. Y. H. F.; formal analysis, J. Y. H. F. and T. T. L.; writing‐original draft, J. L. L. T. and J. Y. H. F.; writing‐review & editing, M. L. Y. and W. K. L.; funding acquisition, J. L. L. T; supervision, J. L. L. T. and M. L. Y.; and all authors have read and approved the manuscript.

## Conflicts of Interest

The authors declare no conflicts of interest.

## Supporting information




**Supplementary Table 1**. Isolates used for analysis in this study.

## Data Availability

The draft genome sequence of “*T. vincentii*” OMZ 800, determined in this study, has been deposited at GenBank under the accession SAMN43900079.
